# Impacts of the 1918 flu on survivors' nutritional status: A double quasi-natural experiment

**DOI:** 10.1371/journal.pone.0232805

**Published:** 2020-10-20

**Authors:** Alberto Palloni, Mary McEniry, Yiyue Huangfu, Hiram Beltran-Sanchez

**Affiliations:** 1 Center for Demography and Health of Aging, University of Wisconsin-Madison, Madison, WI, United States of America; 2 Population Research Center, University of California at Los Angeles, Los Angeles, CA, United States of America; University of Botswana, BOTSWANA

## Abstract

Robust empirical evidence supports the idea that embryonic and, more generally, intrauterine disruptions induced by the 1918-flu pandemic had long-term consequences on adult health status and other conditions. In this paper we assess the 1918-flu long-term effects not just of *in utero* exposure but also during infancy and early childhood. A unique set of events that took place in Puerto Rico during 1918–1919 generated conditions of a “double quasi-natural experiment”. We exploit these conditions to empirically identify effects of exposure to the 1918 flu pandemic and those of the devastation left by an earthquake-tsunami that struck the island in 1918. Because the earthquake-tsunami affected mostly the Western coast of the island whereas early (*in utero* and postnatal) exposure to the flu was restricted to those born in the interval 1917–1920, we use geographic variation to identify the effects of the quake and timing of birth variation to identify those of the flu. We benefit from availability of information on markers of nutritional status in a nationally representative sample of individuals aged 75 and older in 2002. We make two contributions. First, unlike most fetal-origins research that singles out early nutritional status as a *determinant of adult health*, we hypothesize that the 1918 flu damaged the nutritional status of adult survivors who, at the time of the flu, were *in utero* or infants. Second, we target markers of nutritional status largely set when the adult survivors were infants and young children. Estimates of effects of the pandemic are quite large mostly among females and those who were exposed to the earthquake-tsunami. Impacts of the flu in areas less affected by the earthquake are smaller but do vary by area flu severity. These findings constitute empirical evidence supporting the conjecture that effects of the 1918 flu and/or the earthquake are associated not just with disruption experienced during the fetal period but also postnatally.

## Introduction

The Spanish flu virus of 1918–19 is an example of a perfect storm: like HIV and unlike seasonal influenza, it was highly lethal but, unlike HIV and like other influenza, it was rapidly and efficiently spread [1–5]. The combination of these two traits made the pandemic one of the deadliest in human history [6, 7]. As in the rest of the world, the A/H1N1 influenza in Puerto Rico was characterized by its unique temporal sequence, peculiar age pattern, and case morbidity and lethality [8–10]. Jointly, the age pattern of incidence and morbidity and mortality levels, created unfavorable conditions for all but especially for women of childbearing ages and those who were pregnant at the time or who had recently given birth [11, 12]. These conditions may have compromised not just fetal growth but also infant and young children' health, both highly dependent on maternal health status and parental care.

As if the onslaught of the flu had not been enough, on October of 1918, precisely when the pandemic was gathering force during its second, most lethal wave, a strong earthquake (the San Fermin earthquake) struck the Western part of the island. This was immediately followed by a tsunami, two major aftershocks in a two-month interval following the earthquake, and multiple smaller ones spread over the subsequent year or so [13].

Research on the lasting effects of the 1918 pandemic are strengthened by the fact that the event can be considered a quasi-experiment: it was unexpected, difficult to avoid and, in most cases, there were no contemporaneous exogenous events that could have produced similar outcomes [8, 11]. The Puerto Rican earthquake was also unexpected, hard to avoid in areas struck by it and, asides from the flu pandemic, unaccompanied by other major events that could have scarred even more an already vulnerable population. Thus, in one stroke, an unlikely combination of two events handed us conditions of a unique double quasi experiment.

This paper departs somewhat from others on effects of 1918 flu pandemic. First, it seeks to shed light on a rather unexplored dimension of the 1918 pandemic, namely, its effects on *markers of nutritional status* of individuals who were exposed to it *in utero* or during infancy. With the exception of one study [12], we know of no other attempt to investigate such an association. Analyses of impacts on the nutritional status of 1918 flu survivors require a focus on mechanisms that could disturb physiological growth and developmental processes during infancy, early childhood and even early adolescence, not just those that operate *in utero*. It is known that embryonic and, more generally, intrauterine disruptions influence neural development (brain tissue), metabolic balance (pancreas, liver), nephron growth (kidneys and blood pressure regulation) or lung and heart functioning [14–17]. In addition, embryonic and fetal development is also about growth of cartilage, bone, and muscle tissue, all of which are implicated in subsequent postnatal physical development [18]. Furthermore, impairment of growth processes that occur during the fetal period can be aggravated if postnatal conditions deteriorate. Thus, fetal growth could be compromised when pregnant mothers experience illnesses and are exposed to extreme stress.

In addition to these pre-natal mechanisms, the flu and the earthquake separately and jointly must have triggered conditions that, in all likelihood, disrupted processes of growth and development that take place during the first year of life and even later, during early childhood. With the benefit of hindsight that the COVID19 pandemic permits, we know that this must also have been the case in 1918 as infant and child malnutrition in low—to middle -income countries today are expected to be severely affected with lasting future consequences [19]. The social and economic aftermath of the 1918 flu must have borne close resemblance to the devastation being caused by COVID19 and the interventions implemented to halt the spread of the virus: sharp declines in household income, increases in poverty and destitution, lack of access to social, health and other protective services, and massive disruption of food supplies and other resources. But there is more: when, due to illnesses or death, mothers cannot breastfeed normally, are unable to provide adequate maternal care, proper nutrition, grooming, and hygiene, early growth and development could go astray. Furthermore, when infants and children are exposed to subsequent, lasting adverse environmental and material conditions, catch-up growth may be a non-starter [20, 21]. If, in addition, an earthquake-tsunami struck some areas, the destruction there could only have been made worse. For these reasons, it is important to assess not just the 1918 flu 's impacts of in *in-utero* exposure, but also those generated by adverse postnatal conditions.

Second, we build the case on a unique quasi-experimental research design, a product of the occurrence of two simultaneous events, one involving *timing of exposure* (to the flu and the earthquake) and the other *geography of exposure* (areas with different flu severity and differentially affected by the earthquake-tsunami). We aim to show that the flu pandemic and the earthquake-tsunami combine to generate impacts that neither of these events could have produced separately and are strongly associated with both gestational and postnatal exposures.

### Early physical growth debilitation and its long run consequences

Human physical growth depends on early embryonic and fetal events, maternal exposures (including stressors), maternal health status, and parental effects, including maternal capacity to nourish during the fetal and postnatal stages [22–24]. Of particular importance is the length and intensity of breastfeeding [25–27], protection from infections and parasitic diseases [28], recovery from illness [29, 30] and reduction of environmental stressors [31]. These parental effects are strongly associated with maternal (and paternal) health status, household (family) environments and access to resources.

#### Embryonic and fetal growth

By and large, fetal nutrition depends on maternal diet and placental capacity to deliver nutrients (including oxygen, fat, proteins, hormones, SCFA) [32]. It is well-known that maternal nutritional status influences the entire process of fetal development and can have strong impacts of the infant's subsequent growth [33]. It is also known that poor maternal health status can derail the normal course of a pregnancy and complicate delivery. In particular, maternal infections during pregnancy could compromise normal fetal development and the ultimate effects depend on both the timing of infections, their intensity, and duration. These effects are associated with inflammatory responses induced by the infections themselves. In addition to the potentially fetal organogenic damage associated with the flu-related cytokine storms [2, 34], bouts of hyperthemia induced by the inflammation can also lead to deleterious outcomes, including miscarriages, premature labor, stillbirths, congenital anomalies, and growth restrictions [35–37]. The latter are a result of irregularities of the physiology of bone and muscle tissues formation. As is known, bone develops from embryonic mesoderm and proceeds by ossification of cartilage tissue formed from mesenchyme. In addition to bone formation, hyperthermia can also affect limb myogenesis as it disrupts and delays the involvement of several crucial regulatory factors. Jointly, dysregulation of bone and muscle tissue formation can compromise normal physical growth [37].

#### Early and late infant development

Because of mother’s milk properties, intensity and length of breastfeeding is of crucial importance for an infant’s early growth, particularly during the first 6 months of life [38, 39]. Aside from its beneficial nutritional properties [40], breastmilk contains important compounds that strengthen infants’ immune response and act as a shield to reduce risks of viral, bacterial, and parasitic diseases [41]. Most infant infections and parasitic diseases reduce appetite, limit food intake and/or impair the child’s nutrient absorption capabilities [42]. Thus, the combination of illnesses and breastfeeding interruption, cessation, or irregularities during the first 6 months can compromise not only the quality and quantity of nutrients available for early growth but also reduce absorption and metabolization of those available [43]. These disruptions compromise the ability of an organism to satisfy energetic demands to sustain rapid cell division and organ growth and formation during critical periods [31]. Although early growth faltering can be offset by subsequent catch-up growth phase, this will not take place in the absence of material conditions that can sustain rapid growth and maturation [20, 44]. In populations with widespread poverty and vulnerable maternal health status, the process of catch-up growth may never get off the ground and children who could have benefitted from it will fail to attain physical growth milestones [45].

#### Gender differentials in early growth and development

There are important differences between female and male physical growth and development trajectories [46]. Should one expect that responses to insults are also different? Why should this be the case? There are three sets of factors that can cause gender differentials, one promoting stronger effects among females and the other generating stronger effects among males. First, male embryos are known to be more vulnerable than female embryos [18]. This implies that an early sieve operates to remove some of the vulnerable male embryos even in the absence of exogenous shocks. If, in addition, conditions under which growth and development take place turn negative during critical periods, male embryos and fetuses that could have experienced more serious post-natal developmental problems will never be born. Furthermore, it is well-known that male infants experience higher mortality than female infants [47]. Excess infant mortality can operate as a second sieve disproportionally selecting out more frail male births. Thus, it is quite possible that selection forces alone result in stronger effects among females who are, on average, subject to weaker early selection pressures.

However, a second set of factors could amplify effects among males and attenuate them among females. In many low- to middle-income countries, in the past and even more recently, there was pervasive male child preferences that, despite not resulting in outright child neglect, biased the flow of scarce parental resources toward male children [48, 49]. It is plausible to conjecture that under added sources of stress induced by exogenous shocks, male child preferences could have more dire consequences among females than during normal periods, particularly among girls who are more vulnerable to begin with. This is a source of selection that should attenuate effects of early insults among females since those who survive them are of lower than average frailty.

Finally, an alternative explanation that does not rely on selection arguments has to do with gender differentials in growth and development. It has been known for quite some time [50] that male physical growth stretches over a longer period of time than female physical growth. This increases males’ window of opportunity for replenishment and recovery from past interruptions of development and growth. If this is so, males who survive to adulthood could have benefited from these added opportunities and be less likely to carry through life scars associated with very early insults.

Given the nature of our data, it is impossible to sort out between these various forces that combine to produce observed gender differential in responses to the flu and earthquake.

#### Long lasting effects of the flu

The above considerations lead us to hypothesize that exposure to the flu during two critical periods, e.g in utero and/or during infancy, must have had non-negligible influences on early nutritional status and should be reflected in poor adult markers of physical growth. By the same token, exposure to stresses and material deprivation brought about by the earthquake-tsunami could have disrupted embryonic, fetal and postnatal growth and, as consequence, facilitated growth faltering and attainment of substandard markers of physical growth. Furthermore, as did happen in other populations, the effects of the flu are likely to be stronger among those who experienced the pandemic in areas more severely affected by it [8, 11]. Finally, both *in utero* and postnatal vulnerability to the flu was likely augmented by conditions associated with the earthquake [44]. If so, we should find that the impact of the flu among individuals exposed to the pandemic *in utero* or during the first year of life) and individuals exposed later in childhood or adolescence) is larger among those born in areas struck by the earthquake-tsunami (e.g. exposed to the earthquake) than among those born elsewhere in the island. To test these hypotheses, we use a study design that relies on geographic identification of local areas (municipio of birth) classified by flu and earthquake severity. We use dates of birth to assess exposure during critical periods. Table 1 is a representation of the design.

**Table 1:** Stylized representation of study design.

		Municipio of birth			
		Flu Severity		Earthquake Severity	
		Low	High	Low	High
	**Full exposure**				
	(Born 1/2018-12/1919)	A	B	C	D
**Exposure**					
	**No exposure**				
	(Born before 1/2018-after 12/1919)	E	F	G	H

Notes

(i) We use both a dichotomous (Low, High) and a trichotomous (Low, Moderate, Severe) classification of flu severity (see Methods section).

(ii) The severity of the earthquake is gauged by distance to epicenter and we classify municipios as affected (high severity of earthquake) and non-affected (low severity of earthquake) according to distance from epicenter (see Methods section).

(iii) The exposure contrast between cells A-D, on one hand, and cells E-H, on the other, is associated with a cohort' s timing of birth and the conditions to which they were exposed during two critical period (in utero and infancy). Birth cohorts belonging to these cells are at risk of developing growth problems whereas those belonging to cells E-H are not. The contrast between cells A and B, on one hand, and C and D, on the other, is associated with severity of the event, namely, severity of the 1918 flu and "severity" of earthquake (municipios that suffer the earthquake vs municipios that were less affected or not affected at all). There are also contrasts between birth cohorts that belong to mixtures of municipios of type A or B and C or D. Thus, those exposed individuals belonging to cohorts born in municipios that are simultaneously of type B and D are expected to suffer the most. We expect no such contrasts across birth cohorts born in municipios of type E-H.

## Materials and methods

### Data

**Fig 1:**
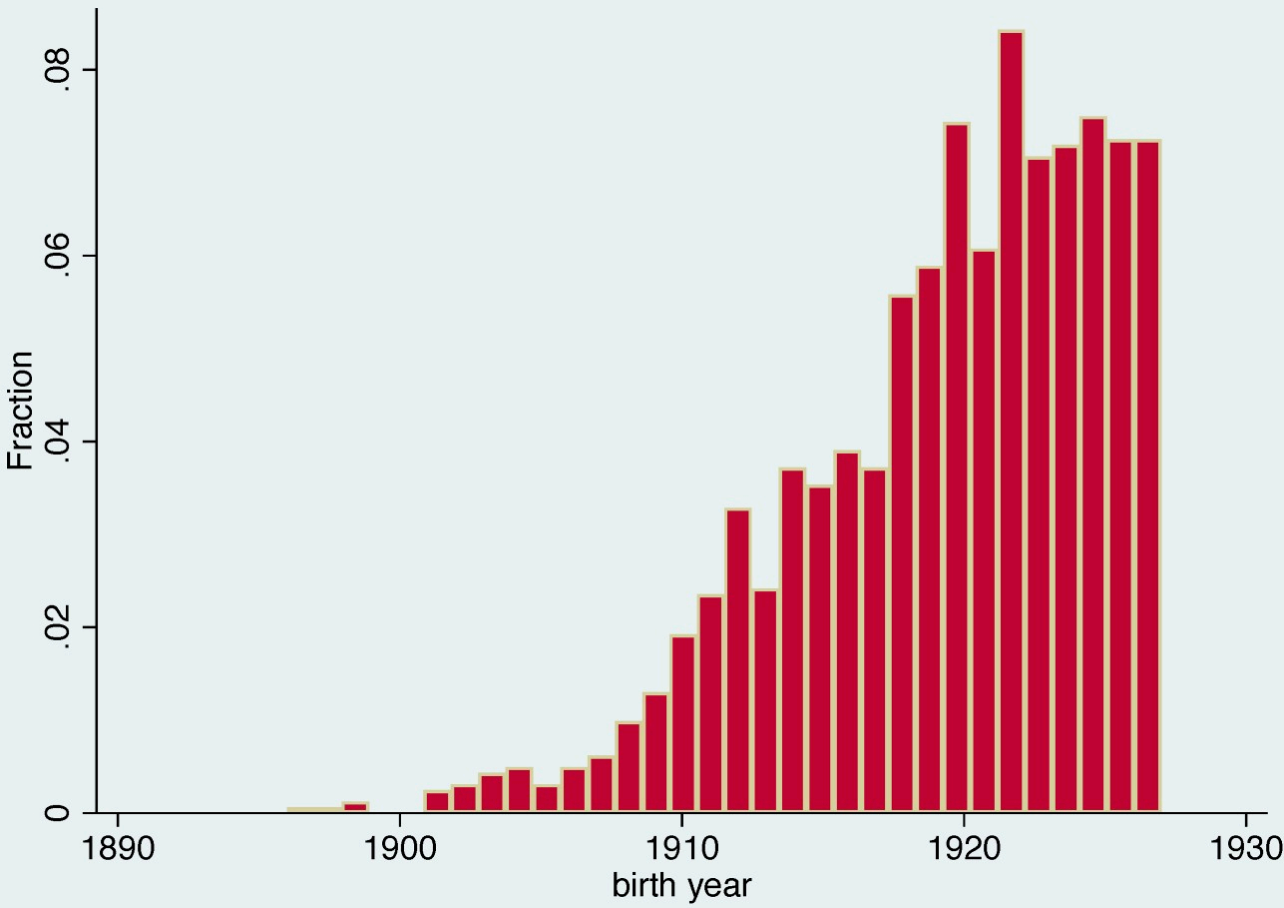
Distribution of year of birth.

We use PREHCO (Puerto Rican Elderly Health Conditions) data base [51]. PREHCO is a two-wave panel of the non-institutionalized Puerto Rican population aged 60 and over and their surviving spouses. The study uses a multistage, stratified sample of the elderly population residing in Puerto Rico in the year 2002 with oversamples of regions heavily populated by people of African descent and of individuals aged over 80. A total of 4,293 in-home face-to-face target interviews were conducted between May 2002 and May 2003 and a second wave data were collected during 2006–2007. The overall response rate was 93.9%. Our analyses use a subpopulation aged 74+ at the time of first interview, e.g. those born between 1896 and 1927. The total sample size is 1,613 observations, 956 of them females. About 30 percent of the sample were born on or before 1917 and 11 percent between years 1918 and 1919. A histogram of the distribution of year of birth is in Fig 1 and a summary of key statistics is in Table 2.

**Table 2:** Summary of selected sample statistics.

Variable		Frequency	Mean	SD
Age				
	74–79	0.38	-	-
	80–84	0.32	-	-
	85–89	0.17	-	-
	90+	0.12	-	-
	All	-	82	5.7
Gender				
	Males	0.41	-	-
Poverty				
	Born in poor municipios	0.4	-	-
Flu Severity				
	Born in high severity municipios	0.29	-	-
Exposure				
	To 1918 Flu (Exposure_Flu = 1)	0.09	-	-
	Earthquake (Exposure_earthquake = 1)	0.14	-	-
Knee height (cms)		-	46	4.8
Height(cms)		-	155	9.7
Education (years)		-	6.7	4.9
Total N		1613	-	-
Total missing anthropometry		283	-	-
Effective sample size		1330	-	-
Cases exposed (Born 1918–1919)			142	
Cases not exposed Born 12/1917& 1918/after12/2019			1471	
Cases in severe earthquake municipios			471	
Cases in severe flu municipios			230	

* With the exception of knee height and height, all statistics are computed using the total number of observations (1613).

Puerto Rican birth cohorts born before 1927, were exposed to mortality experiences similar to those in Latin American countries. Their infant and early child mortality levels correspond to life tables with life expectancies at birth not exceeding 35 years and are in the range of 180–220 per 1,000 births. Up until 1950 and during their late adolescence and early adulthood, these birth cohorts' mortality conditions are embedded in life tables with life expectancy at birth of around 45–50 years. Mortality improvements relative to early infancy and early childhood largely originate in eradication of parasitic and vector born infectious diseases (period 1910–1930) as well as to increasing standards of living that marked the period 1900–1940. Finally, their post-1950 mortality experience is heavily influenced by the introduction of chemotherapy (antibiotics, sulfa) and vaccinations as well as by increased prevalence of modern chronic illnesses, including Type II diabetes, heart disease and cancer [52] Life expectancy at birth between the years 2000 and 2005, the period during which PREHCO was fielded, was of the order of 78–79. These cohorts' life expectancy at ages 60 is of the order of 18–20 years. In summary, the birth cohorts we study experienced highly heterogeneous mortality conditions throughout their lifetime, ranging from severe during their early childhood years to mild and lenient in later life.

### Measures

#### Flu exposure

In contrast to other studies of the 1918 flu, we identify a wider period during which exposure is assumed to have taken place and, in addition to the fetal period, include time intervals during which post-natal care may have been disrupted as a consequence of the epidemic and/or the earthquake. If, as it is most likely, the post-natal mechanisms are also relevant for outcomes other than physical growth markers (adult health, mortality, cognition, educational attainment, etc…), studies that ignore them will underestimate the total effects when using a restrictive definition of exposure for some "treated" cases will be assumed to be "controls". To capture the extended exposure including gestational and post-natal exposures we define a dummy variable attaining the value 1 if an individual's birth is reported to have taken place during 1918 or during the first six months of 1919 (See Appendix for alternative definitions of exposure). This indicator is a compromise between preservation of the ability to assign effects to *fetal and post-natal exposure according to timing and duration* and sample size constraints.

#### Flu severity

Because we lack information on the incidence and case fatality of the pandemic in Puerto Rico, we follow past research and create a proxy indicator of flu severity using the excess total mortality registered during the flu period [1]. To construct an index of severity we consider total mortality during the two years period 1918–1919 for each of the 76 municipios, the smallest administrative units in Puerto Rico, estimate expected deaths using age-specific mortality rates during 1918 in the US, and then compute the ratio of mortality rate observed in a municipio to the observed rate [53]. The resulting quantity is an indirectly standardized mortality ratio, a conventional index computed when information of age specific death rates is absent. We classify as high severity all municipios above the 90th centile of the severity index distribution. Those between the 50th and 90th centiles are classified as intermediate severity and the remaining as low severity (See S1 Fig).

#### Earthquake-tsunami exposure

Exposure to earthquake-tsunami is assessed according to the timing of birth of a cohort and municipio of birth. We consider that a birth cohort’s early conditions may have been affected by the earthquake if the timing of birth falls within the same window defined as critical for the flu (see above). Thus, to capture extended exposure including gestational and post-natal exposures we use the same dummy defined to flag flu exposure.

#### Earthquake severity

We classify municipios of birth into three groups depending on the severity of the earthquake: (i) most severe (all municipios in the West coast of Puerto Rico, (ii) mild (all municipios to the East and immediately adjacent to those classified as severe and (iii) not severe, (the remaining municipios) (see S4E Table for municipio membership by classes In what follows we use a 0/1 dummy variable to flag municipios in group (i).

#### Municipio poverty levels

To assess poverty levels in municipios of birth we adopt the classification constructed by Clark [54]. Municipios were grouped into three classes according to their population size, assessed value, and government income. A total of 25 municipios are either in the wealthiest or an intermediate class and the remaining ones are in the poorest category. In this paper we use a 0/1 binary indicator to contrast the poorest and the remaining municipios. When using this indicator in models with no fixed effects we verify that the indicator behaves as expected, namely, it is strongly and negatively correlated with both knee height and height. The models we discuss in this paper, however, only rely on a fixed effects formulation and the impact on municipio of birth poverty levels is absorbed by the fixed effects. Thus, an important set of confounders associated with early poverty are neutralized in the fixed effect model.

#### Markers of early nutritional status: Knee height and adjusted height

We use PREHCO's anthropometry module for the assessment of height and knee height [55]. To attenuate biases due to skeletal compression, we adjust height measures using estimates of compression by gender and age observed in a sample of individuals who were followed for a long period of time (see S2 Fig) [56]. The magnitude of the adjustments is considerable and, if anything, they will lead to overcorrection and downward biases of effects on height. On the other hand, knee height is also a marker of early nutritional status and is unaffected by skeletal compression. Other outcomes are frequently studied in the literature on the 1918 pandemic. Among them is BMI. We do not examine these here since our interest is on markers of early nutritional status and neither BMI nor any of many indicators available in the survey are suitable.

### Models

We use OLS regressions with municipio fixed effects and treat adjusted height and knee height as continuous variables. We also estimated SUR, models to account for correlation between knee height and height. Results from these models are available upon request. Although there are some differences between SUR and OLS estimates (standard errors of regression coefficients), the differences are minor and do not affect inferences. We also estimated biprobit models using binary indicators for knee height and height. The drawback of these models is that they depend on arbitrary cut points. In all cases we use our preferred measures of exposures, namely, *Exposure = 1* to flag birth cohorts born during the critical period defined before and to distinguish them from birth cohorts born before and after such critical period (*Exposure = 0)*. We consider severity of flu using two alternative indicators. The first is a single dummy variable (*Severity_flu*) that distinguishes between municipios where the flu is considered to be severe from those in which it was mild. The second consists of two dummies that identify municipios with severe and intermediate severity. A dummy variable, *Severity_earth*, serves to distinguish municipios which were severely affected by the earthquake from all others. In addition, the model contains first, second and third order interaction effects. The specification for outcome k (k = 1 for knee height and k = 2 for height) is

$$Z_{ki}=\alpha_{ki}+\beta_{k}\;*\;C_{i}+\gamma_{k}*Exposure_{i}+\varphi_{k}*Severity\_flu_{i}+\lambda_{k}*Severity\_earth_{i}+\theta_{k}*Exposure_{i}*Severity\_flu_{i}+\delta_{k}*Exposure_{i}*Severity\_earth_{i}+\mu_{k}*Exposure_{i}*Severity\_flu_{i}*Severity\_earth_{i}+\varepsilon_{ki}$$ (1)

where Z_ki_ is the outcome k for individual i. **C**_i_ is a vector of control variables that includes years of education, year of birth, and a dummy variable for gender. This regression formulation is a difference-in-difference model that seeks to identify (i) differences in the impact of the flu by timing of exposure between high and low flu severity areas and (ii) differences in the impact of the earthquake by timing of exposure between areas affected by high and low severity of the earthquake-tsunami and, finally, differences in the effect of exposure between areas affected by both high severity of flu an earthquake and those only impacted by one of these. Exposure_i_ is a 0/1 variable for exposure to flu/earthquake. Severity_flu_i_ is a 0/1 variable for flu severity in the municipio of birth; in some models we used a more fine-tuned classification and employed two dummies to distinguish municipios with high, moderate(intermediate) and low severity. Severity_earth_i_ is a 0/1 variable for earthquake severity in the municipio of birth, and ε_ki_ is an idiosyncratic error. In turn, the parameters for the equation of outcome j are a constant, α_k_, and a vector of effects associated with controls, **β**_**k**_. The effect of exposure, γ_k_, reflects the combined impacts of flu and earthquake exposure for cohorts born during the critical period. The parameter θ_k_ measures the difference of effects of flu exposure between those born in high and low severity municipios whereas the difference of effects of exposure between those born in municipios affected by the earthquake and those born in the remaining municipios is, δ_k._ Finally, μ_k_ is a measure of the excess impact of exposure during the critical period among individuals born in municipios with high severity flu and affected by the earthquake relative to those also exposed but in areas of low flu severity and not affected by the earthquake. To assess differentials by gender we also introduce interaction terms involving the 0/1 binary variable for gender (female = 1 for females), timing of exposure, and flu and earthquake severity.

A few remarks are important. First, our models *do not* include a control for age as there is no relation between markers of nutritional status and age. Second, additional controls were tried but discarded since they did not change results. Third, all our initial models included a linear term for birth year to account for secular trends in height and knee height as well as to purge out effects of other time variant factors related to birth cohorts. Since estimates from models that did include these covariates are no different from those that did not, we only discuss results from the latter. Fourth, we estimate models with municipio fixed effects. This implies that the main effects of earthquake and flu severity, as well as other variables at the municipio level, are absorbed by the fixed effects. Finally, we estimate models with adjusted variance-covariance matrices of errors to relax assumption of independence of observations and requiring only.

#### Model justification and interpretation

Estimates from the above model can be interpreted causally only if some conditions are satisfied. First, other than the earthquake and flu, there are no concurrent phenomena that could influence outcomes. Second, the occurrence of earthquake and flu are uncorrelated. Third, unmeasured cohort conditions that influence the outcomes of interest vary continuously between cohorts. Finally, selection due to differential survival among those exposed and non- exposed is minor.

The first condition is likely to be met since, to our knowledge at least, other than being very marginally affected by military mobilization and disruptions caused by WWI, Puerto Rico was not affected by other simultaneous large-scale disruption. The second condition is also likely to be met. However, our assessment of flu severity relies on estimated excess deaths during the flu period. Because the earthquake also contributed to excess deaths, at least in areas severely affected by it, the severity indicator could introduce a correlation between the two events. Since in most of our analysis we rely on a coarse binary indicator for severity, this correlation is unlikely to influence results. The third condition refers to the possibility than unmeasured conditions associated with the nutritional status of cohorts born at the time of the flu and earthquake, changed abruptly as a consequence of these shocks themselves and/or some concurrent event. For example, if draftees to WWI armies were disproportionately drawn from middle income classes, the composition of births by class origin during that period suddenly changed. Since social class of origin is strongly associated with nutritional status, these birth cohorts would be composed by individuals who are at higher risk of manifesting deficits in adult markers, independently of the flu and the earthquake. But, the population of Puerto Rican WWI draftees from middle to high classes in US armies must have been insignificant since the total number of Puerto Rican recruits was in the hundreds, not thousands.

The final condition is that there is no selection due to differential survival of individual exposed and non-exposed to exogenous shocks. Because conditions that determine poor early nutritional status also increase child and adult mortality risks, it is quite likely that selection in our sample of older adults will induce to downward biases on estimates of effects of exposure to flu and earthquake. In S1 File we provide a rough assessment of the potential magnitude of these biases.

## Results

We first discuss results from baseline models for knee height and height that only include exposure during the critical period (and years of education as a control variable). We then examine whether there are differences in the effects of exposure across areas with contrasting flu severity. This is followed by a review of models that also include a dummy variable for earthquake and higher order interaction effects. These models enable us to assess whether the pandemic and the earthquake combine to induce impacts of larger magnitude than either event separately. Individual regression coefficients are displayed in Tables 3–6 (Table 7 is a compact summarizes of estimates of *total* (net) effects.)

**Table 3:** Baseline models.

Variables	Knee height	adjHeight
Exposure	-1.24*	-0.006
	(0.61)	(1.10)
Female	-3.49*	-11.56*
	(0.27)	(0.44)
Female x Exposure	-0.09	-2.64*
	(0.95)	(1.39)
Years education	0.08	0.26
	(0.03)	(0.048)
Constant	47.08	165.26
	(0.29)	(0.31)
R-squared	0.15	0.42
Between var	2.4	2.98
Within var	4.11	4.61
Observations	1290	1265

Standard error in parentheses

** p < .01

*p < .025

^p < .05

**Table 4:** Models with flu severity.

Variables	Knee Height	adjHeight
Exposure	-1.02^	0.28
	(0.67)	(1.31)
Exposure x Severity_flu	-0.86	-1.3
	(1.46)	(2.58)
Female	-3.58**	-11.66**
	(0.3)	(0.59)
Female x Severity_flu	0.27	0.32
	(0.60)	(0.79)
Female x Exposure	0.14	-2.88*
	(1.04)	-1.42
Female x Exposure x Severity_flu	-0.67	1.11
	(2.1)	(3.79)
Years education	0.084*	0.26**
	(0.3)	(0.048)
Constant	47.07	165.26
	(0.29)	(0.32)
R-squared	0.15	0.42
Between var	2.4	2.98
Within var	4.11	6.93
Observations	1290	1265

Standard errors in parentheses

** p < .01

*p < .025

^p < .05

**Table 5:** Models with flu severity (females).

Variables	Knee Height	adjHeight
Exposure	-0.8	-2.81**
	(0.73)	(0.82)
Exposure x Severity_flu	-1.84*	-3.24
	(0.77)	(2.75)
Years education	0.087*	0.27**
	(0.041)	(0.064)
Constant	43.46	153.68
	(0.25)	(0.7)
R-squared	0.015	0.058
Between var	2.25	3.39
Within var	4.04	6.65
Observations	758	744

Standard errors in parentheses

** p < .01

*p < .025

^p < .05

**Table 6:** Models with flu and earthquake severity.

Variables	Knee Height	adjHeight
Exposure	-0.95	0.24
	(0.65)	(1.36)
Exposure x Severity_flu	-1.26	-4.84
	(2.23)	(4.19)
Exposure x Severity_earth	-3.75**	0.96
	(1.16)	(1.8)
Exposure x Severity_flu x Severity_earth	4.72	5.12
	(2.95)	(4.64)
Female	-3.64**	-11.68**
	(0.33)	(0.62)
Female x Severity_flu	0.11	0.64
	(2.23)	(1.04)
Female x Severity_earth	-1.18	-3.24
	(1.85)	(3.93)
Female x Exposure	0.029	-2.75*
	(1.07)	(1.47)
Female x Exposure x Severity_flu	2.17	5.08
	(3.09)	(6.19)
Female x Exposure x Severity_earth	-3.72	-3.24
	(3.53)	(3.93)
Female x Exposure x Severity_flu x Severity_earth	-10.28**	-4.39
	(4.72)	(7.33)
Years education	0.083**	0.27**
	(0.031)	(0.05)
Constant	47.07	165.25
	(0.29)	(0.32)
R-squared	0.16	0.42
Between var	2.45	2.99
Within var	4.11	6.94
Observations	1290	1265

Standard errors in parentheses

** p < .01

*p < .025

^p < .05

**Table 7:** Synthesis of results.

MODELS		KEY FINDINGS		TOTAL EFFECTS (females only)	
		Knee height	Height	Knee height	Height
**Baseline model with exposure only (I)**	Effect of exposure only	Significant	Significant	Obs = -1.33	**
				F* value = 3.13	Obs = -2.65
				prF_o_ >.F* =. 08	F* value = 10.4
					prFo>F* = .0020
**Models with exposure and flu severity (II)**	Effects exposure only (a)	Exposure: not significant	Exposure: Significant	Obs = -.88	**
				F* value = 1.56	Obs = -2.60
				PrF>F* = .22	F* value = 9.87
					prFo>F* = .0025
	Total effects exposure in high flu severity areas (b)	Exposure and high flu severity: not significant	Exposure and high flu severity: Not significant	Obs = -2.14	Obs = -2.48
				F* value = 2.08	F* value = 1.65
				prFo>F* = .15	prFo>F* = .20
**Models with exposure and flu severity (females only) (III)**	Effects of exposure only (a)	Not significant	Significant		**
				Obs = -80	Obs = -2.50
				F* = 1.18	F* = 8.72
				prFo>F* = .28	pfFo>F* = .004
	Total effects of exposure in high flu severity areas (b)	Significant	Significant	**	**
				Obs = -2.64	Obs = -6.05
				F* = 79.13	F* = 5.38
				prFo>F* = .000	prF>F* = .024
**Models with exposure and flu and earthquake severity (all) (IV)**	Effects of exposure only (a)	Not significant	Not significant		**
				Obs = -.92	Obs = -2.50
				F* = 1.59	F* = 8.72
				prFo>F* = .22	prFo>F* = .004
	Total effects of exposure in high flu severity areas (b)	Not significant	Not significant	Obs = -.37	Obs = -2.26
				F* = .07	F* = .53
				prFo>F* = .79	prFo>F* = .47
	Total effects of exposure in high earthquake severity areas (c)	Not significant	Not significant	Obs = —68	Obs = -4.78
				F* = .86	F* = 2.10
				pfFo>F* = .86	prFo>F* = .16
	Total effects of exposure in high flu and earthquake severity areas (d)	Significant	Significant	**	**
				Obs = -5.68	Obs = -8.92
				F* = 24.03	F* = 3.83
				pfFo>F* = .000	pfFo>F* = .05
**With exposure and flue and earthquake exposure (females only)**		Results similar to those from model estimated with the entire sample		NA	NA

Notes:

(i) Column 1 identifies the model; column 2 identifies the effect of interest; columns 3 and 4 contain brief descriptions of results; columns 5 displays estimates of exposure only; column 6 displays estimated total (net) effects and F-test statistics

(ii) Cells with two stars flag findings with statistical support; cells with one star flag marginal support; cells with no stars identify findings with no support.

### The flu: Models for knee height and height including exposure during critical period and flu severity

#### Baseline models

Table 3 displays estimates of coefficients for models with knee height and height as dependent variables, control variables, and the additive and interaction effect of gender. As assessed by the overall value of R-square, the model for height fits much better than the one for knee height (.42 vs .15). The reason for this is that gender is a much stronger predictor of height than of knee height. In any case, the goodness of fit of these models is quite good for this kind of individual level data. Note that the coefficients for the single control variable, years of education, is very large and in the expected direction. This result is reassuring because it confirms that educational attainment reflects early conditions that influence markers of nutritional status. Note that because individual adult educational attainment itself could be influenced by the pandemic, the relation between education and markers of nutritional status could also be spurious. Second, the effects of exposure during the critical period (*Exposure)* are in the expected direction although it is only statistically significant in the model for knee height where the magnitude of the coefficient is close to twice the standard error. The reduction in knee height among those exposed during the critical window is about 1.24 cms or 4 percent of the mean value in the sample and the corresponding regression coefficient is marginally significant (-1.24 (.61)). The magnitude of this effect is slightly smaller than a third of the total effect of gender (-3.49), the covariate that has the largest effect in the model. It should be kept in mind that this estimate mixes the impact of exposure to both flu and earthquake. The total reduction in knee height among exposed females is 1.33 (-1.24+(-.09)) and an F-test reveals this is significant at the .04 level. Third, the model for height reveals a gender differential that penalizes females: the reduction in height among exposed females is of the order of 2.65 cms, about 2 percent of the mean value in the sample, with a coefficient about twice its standard error. An F-test confirms that the total effect exposure on females' height is different from 0 (pr F_o_ > F* = 10.4 is .0020).

Thus, exposure during critical period leads to losses of knee height and height but gender differentials in these losses are only manifested in adjusted height.

#### Models with flu severity

Table 4 displays only estimates of parameters of interest from models that include the interaction term between exposure and flu severity as well as those associated with the gender variable. As in the previous case, the model does not control for earthquake and the effect associated with the variable exposure absorbs both the impact of the flu and the earthquake. Three results stand out. The additive effect of exposure on knee height is properly signed and slightly larger than in the baseline model (-1.02 (.67)) whereas the added impact on females is close to zero (.14 (1.04)). The effect of exposure is larger in the hardest hit regions and leads to a decrease of about .86 cms but the regression coefficient does not pass a standard statistical test (-.86 (1.46). Third, the impact among exposed females in high severity area is weak (-.67 (2.11)) and statistically insignificant.

The model for height leads to somewhat different inferences, however. As in the previous case the additive effect of exposure is small but, unlike the model for knee height, there is an important gender differential as exposed females lose about 3 cms (about 3 percent of the mean) and the regression coefficient estimate is more than twice its standard error (-2.87(1.42)). In fact, an F-test reveals that the *total impact* among females (the additive effect of exposure and the interaction between exposure and gender) is statistically different from 0 (pr F_o_ > F* = 9.87 is .0025). However, there is no evidence of a contrast between impact of exposure in the total population (-1.30 (2.58)) nor among females (1.11 (3.80)).

**Fig 2:**
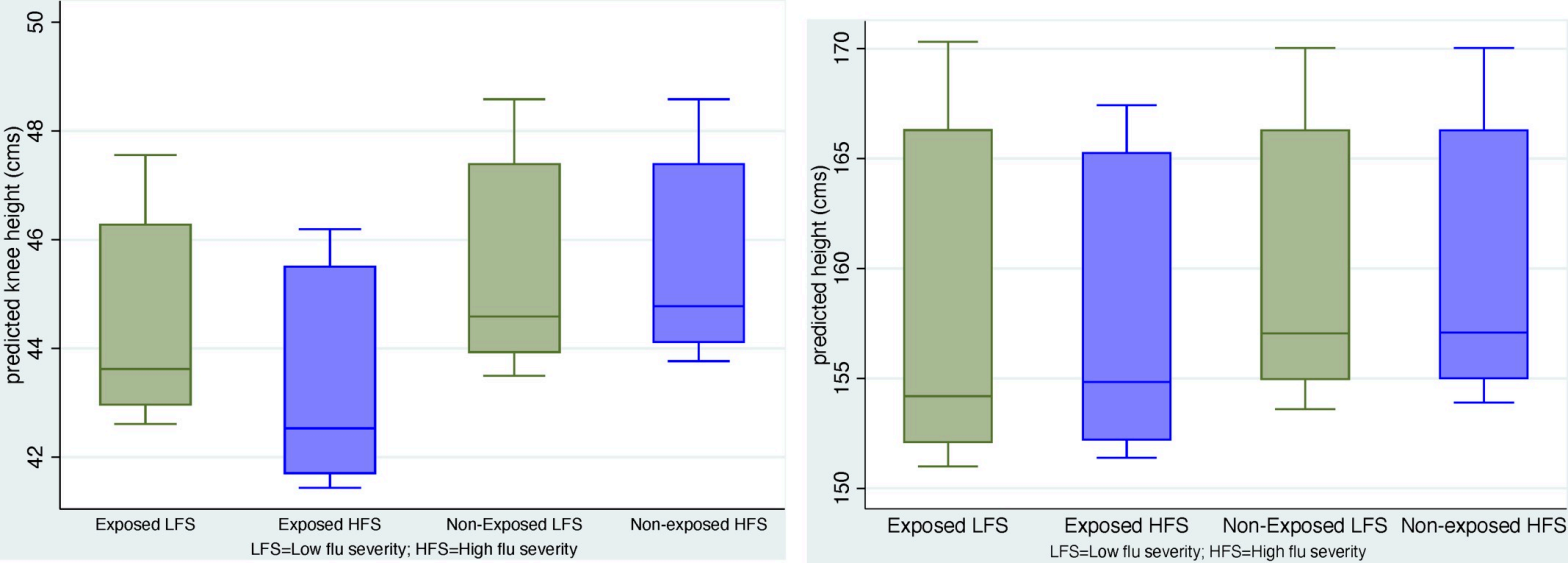
A. Box plots of predicted values of knee height. (Models including flu severity-full sample). B. Box plots of predicted values of height. (Models including flu severity-full sample).

Fig 2A and 2B display box-plots of predicted values of knee height and height for exposed and non-exposed individuals born in high and low flu severity municipios using coefficients from Table 4. The gradient between exposed and non-exposed and between those born in different flu severity municipios is apparent for knee height and of the order of 2 to 4 cms. The contrasts are in the expected direction for height but are of smaller magnitudes.

Because we find important differentials by gender, we also estimated a model with the female subsample only. Although we lose some statistical power, we gain somewhat by reducing the confounding impact of heterogeneity of effects by gender. Furthermore, to fine-tune the indicator for flu severity we use all three classes of severity (small, moderate and severe). Table 5 displays the new models' estimates. These suggest somewhat stronger inferences than allowed by the previous model. In fact, although the additive effects of exposure on knee height is correctly signed but small, the interaction effect associated with the most severely affected municipios are quite large and more than twice their standard errors (-1.84(.77)). Exposed females in areas of high severity lost a total of nearly 2.64 cms in knee height or about 5 percent of the mean in the sample. The F-test indicates that the *total effect* of flu exposure is statistically significant (prF_o_>F* = 79.13 is .0002).

**Fig 3:**
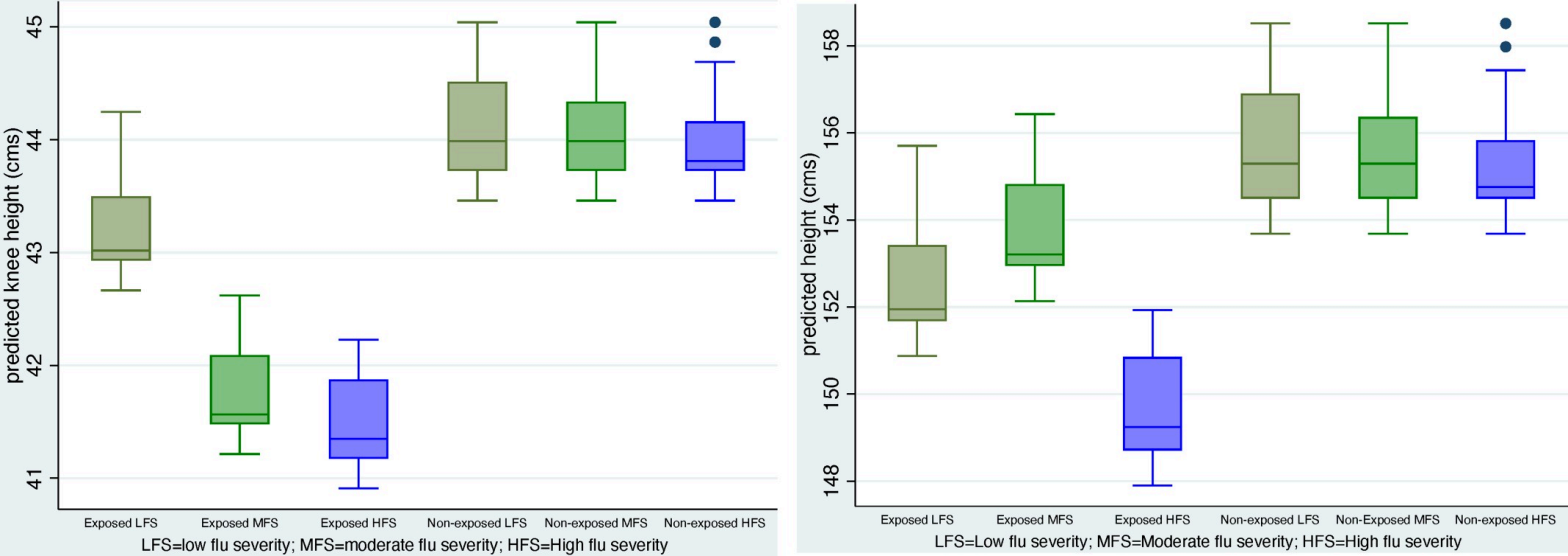
A. Box plots of predicted values of knee height. (Models including flu severity-female sample). B. Box plots of predicted values of height. (Models including flu severity-female sample).

The results for height are even stronger: exposed females in high severity areas lost a total of 6.05 cms (-2.81+ (-3.24)) or about 14 percent of the mean in the sample and both the additive and interaction coefficients are statistically significant. As in the case of knee height, the F test for the total impact on height confirms that the overall impact is important (prF_o_ > F* = 5.38 is .024). Fig 3A and 3B display plots of predicted values of knee height and height by flu severity using coefficients from Table 5. The gradients are sharp and suggest losses of about 1.5 to 2 cms of knee height and 3 to 4 cms of height among those exposed in high flu severity areas (relative to those born in low flu severity areas). Contrasts are large (between 3 and 4 cms of knee height and 5 and 6 cms in height) between those exposed and not exposed.

The observed patterns identified above can be summarized as follows: had we been interested only on the impact of the flu and ignored the concurrent earthquake-tsunami, we would have stopped the analysis here. Our conclusion would have been that females bore the brunt of the flu pandemic and that those born in municipios hardest hit by the flu experienced damage in both phenotypes of interest as they lose as much as 3 to 4 cms of knee height and 5–6 cms of height. However, during the period that the pandemic prevailed in the island, and coinciding with its peak strength, a strong earthquake-tsunami castigated the West costal and proximate interior region of the island. It is then possible that inferences from models that ignore the earthquake attribute to the flu (in high and low severity areas) impacts that might be attributable to the earthquake. This is an example where the joint occurrence of two exogenous shocks, the flu and earthquake, violates one of the prerequisites needed to justify treating the pandemic as a quasi-natural experiment. The only escape from this trap is to estimate models that account for earthquake severity.

Could the estimated influenza effects be confounded with impacts of the earthquake? Were the effects of the flu magnified in areas affected by the earthquake or those of the earthquake magnified in areas of high flu severity?

To answer these questions, we estimated models including the additive and interactive effects of the earthquake. We seek to determine whether (i) there are independent effects of the flu and the earthquake and their relative magnitudes, (ii) there are important synergies between the two events, and (iii) females were singularly affected.

### The flu and the earthquake: Models for knee height and height including flu and earthquake

Table 6 shows results of the full models that include exposure, flu severity, and earthquake severity as well as interaction effects associated with gender. Because the introduction of a new dummy for earthquake and associated interaction effects leads to substantial losses of observations in some cells of the data, we return to use the binary variable for severity of earthquake as identified in Table. The results for knee height are somewhat mixed but they shed light on an intriguing story and are summarized below. The results for height, on the other hand, are weaker although they too reveal important regularities.

#### Knee height

Does exposure during critical windows make a difference? The additive effect of exposure in the model for knee height is properly signed but significant only at .10 level (-.95 (.65)). In contrast, exposure during critical windows among individuals born in municipios more severely affected by the earthquake translates into sizeable and significant effects independently of gender. The estimate of the corresponding regression coefficient implies knee height reductions of the order of 3.75 cms and the associated regression coefficient is significant at the .004 level. However, exposure in high flu severity areas does not appear to lead to important reductions in knee height as the estimated effect is negative, as expected, but does not attain statistical significance (-1.26 (2.23)). Similarly, exposure during critical periods in municipios of high flu severity and also affected by the earthquake does not result in particularly large negative impact. In fact, the opposite appears to be the case as the additive effect of exposure during critical periods in areas affected by the earthquake *and* with high flu severity is positive albeit with a large standard error (4.72(2.95)).

**Fig 4:**
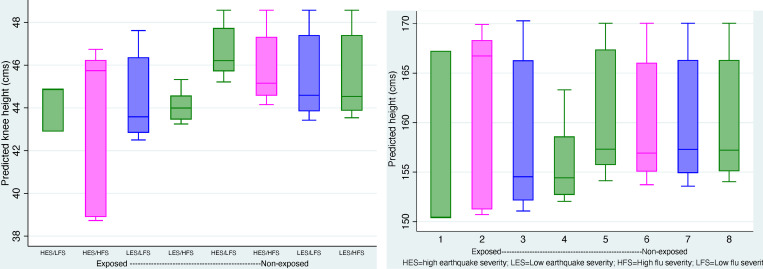
A. Box plots of predicted values of knee height. (Models including flu and earthquake-full sample). B. Box plots of predicted values of height. (Models including flu and earthquake-full sample).

The final problem we investigate is whether the above patterns are different for females. Exposed females in the general population are not affected (2.17 (3.09)). Nor are those exposed and born in earthquake municipios (3.72 (3.53)) or exposed and born in high flu severity areas (2.17 (3.09)). However, exposed females born in areas struck by the earthquake *and* of high flu severity are singularly affected. In fact, we find a sizeable and significant impact among females exposed during the critical period who were born on earthquake *and* high flu severity areas. The estimate of effects for these females implies a reduction of knee height of about -10.28 cms, a figure that is statistically significant despite a large standard error. Because the additive effect of exposure in areas of high flu severity and affected by the earthquake implies an average *increase* in knee height of about 4.72, albeit with a large standard error (2.95), the *total effect* among females is negative and leads to a loss of 5.68 cms. To check the importance of these effects we employ two F-tests. The first corresponds to the null hypothesis that the magnitude of the overall effects of the earthquake and the flu among females is zero, that is, the sum of the effects females' experience by virtue of being exposed during critical periods, being born in areas affected by the earthquake, and with high flu severity is not significantly different from 0. The observed total or net impact (5.68) yields an F = 24.03 (prF_o_ > 24.03 is .0001). The second test seeks to verify the null hypothesis that the difference between effects of females exposed during critical windows and those NOT exposed is 0. The observed value of the test statistic is equal to -6.41 among females exposed and -.73 among those not exposed. The difference translate into an F statistic (21.78) that would be observed in a population where the difference is zero with a probability of less that .0001. These two tests offer empirical evidence confirming that females whose exposure during critical windows took place in areas of high flu severity and affected by the earthquake are most damaged by these events. Fig 4A and 4B are box plots of predicted values of knee height for selected categories of exposed and non-exposed individuals who experienced combinations of the worst conditions. Although there is important within-category variability (particularly in the worst one, namely, among individuals exposed in high earthquake and flu severity areas) the between category gradients are in accordance with the conjectures.

#### Height

The results for height are weaker than those for knee height even for females. Exposure during critical periods by itself does not influence height (.24 (1.36)) nor does it whether it is combined with the earthquake (.96 (1.80)) or high flu severity areas (-4.84 (4.20)) or both (5.12 (4.64)). The last two regression coefficients are large but very noisy, possibly due to small number of cases.

As was the case for knee height, exposed females are more affected as they lose 2.74 cms and the corresponding regression coefficient is marginally significant. Females who were exposed in earthquake areas experience reductions in height but the estimate is too noisy (-3.24 (3.93)). The effect on females who were exposed in high severity areas appear to gain in height but, here again, the regression coefficient is subject to quite a bit of uncertainty (or in high flu severity municipios (5.08 (6.19))).

Finally, and unlike the case of knee height, height reduction among exposed females born in earthquake and high severity areas is large but only half its standard error (-4.39 (7.33)). As in the case of knee height, however, the total impact on exposed females is equivalent to a total loss of about 8.92 cms, and statistically significant (prF_o_>F = .05). The *difference* between impacts on females exposed and those not exposed is 5.93 cms (prF_o_ > F = .0018)

The above results suggest four inferences. First, the flu pandemic alone had important impacts on knee height and height but only among females born in high severity flu areas. Second, the earthquake-tsunami and the flu pandemic combined to induce damage but mostly on females' knee height and somewhat less on females' height. The net effects on knee height are sizeable, translate into reductions of the order of 5.69 cms or about 10 percent of the mean value of knee height in the sample, and are statistically significant from 0. The net effects on height are also statistically significant and equivalent to losses of 10 cms representing about 6 percent of the mean value in the sample. The total effects of female exposure on knee height are two to three times larger among those who were born in municipios with high flu severity and affected by the earthquake.

Table 7 is a synthetic representation of key results for the female subpopulation. The table includes a brief summary of main findings (columns 3 and 4) and estimates of total effects and associated F-statistics (columns 5 and 6). Yellow cells represent findings consistent with initial conjectures. Green cells are associated with weaker evidence and grey cells with little or no empirical support. An examination of yellow cells suggests that female exposure during the critical period led to total losses in knee height and height of the order 1.33 and 2.65 cms respectively (first row of and last two columns of table). Exposure within high flu severity areas increases these losses to 2.63 a 3.44 cms (fifth row and last two columns of the table). Finally, when exposure takes place in areas hit hard by both the earthquake and the flu losses increase to about 5.7 and 9.9 cms respectively (next to last row and last two columns).

### Interpretation and robustness of results

#### A cautionary note on interpretation

The impacts in Table 7 are not trivial and, given the definition of exposure we use here, they are consistent with the idea that it is the combination of perturbations during the fetal *and* the postnatal period that mediate the impacts of the two external shocks that struck this population. These effects are larger than those one gets when using an exposure variable that constrains the critical window to the fetal period only. But, how strongly can we lean on this interpretation of findings? Although the empirical analysis presented above is suggestive and consistent with the initial conjecture there are alternative interpretations.

**Fig 5:**
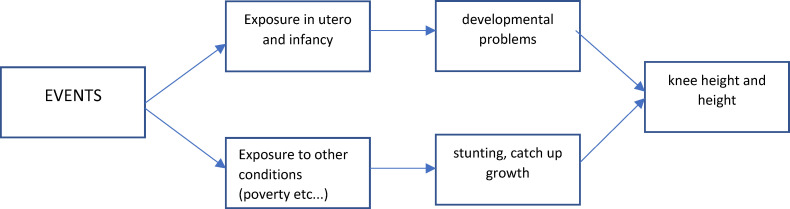
Graphic representation of relations under study.

Human markers of physical growth and development, other than those assessed at birth, are shaped by a multiplicity of factors, from genetic make-up to pre-pregnancy maternal health status to exposures in utero, to conditions encountered in infancy, early childhood and adolescence, and to adult and older adult experiences. Even though the two markers of nutritional status we use here, height and knee height, measure phenotypes whose growth and development dynamics are subject to important constraints that are set early in life, they are also shaped by conditions experienced after the narrow window we flagged here as critical. Furthermore, these conditions are directly or indirectly related to the two exogenous events of interest. Fig 5 is a rendition of the main relations involved. The figure is designed to emphasize that the way to secure evidence supporting a strong interpretation that attributes effects of external events on adverse early conditions *only* is to control for “other factors” also influenced by the events. Although the effects of the latter could very well be accounted for by individual measures (education) and contextual indicators (municipio poverty index), all of which are included in our models, they may be insufficient to purge effects represented by the path in the lower part of Fig 5. Childhood stunting induced by a protracted period of poverty following the earthquake may derail physical growth permanently. If so, the effects we estimate cannot be solely imputed to exposure during the early critical window we focus on here. But, by the same token, catch-up growth in early adolescence, years after the event of interest took place, could conceal effects of exposures in the critical window. These two sets of effects will offset each other, and we will not be able to rank them in importance. For this reason, we argue that it is inappropriate to downplay the mediating role of early conditions. The two markers of physical growth, but less so knee height than height, are influenced by heterogeneous conditions that are experienced in very different windows of time during fetal development (particularly during the last three months of pregnancy), infancy, early childhood and adolescence. Because they involve biological processes that unfold at different stages, they are not perfectly correlated. In fact, the correlation between these two measures ranges from .3 to .6 [57–59]. Also, it is known that birthweight is correlated with adult height and that this association is partially explained by intrauterine conditions and genetic factors [60]. Although we know of no study confirming this, it must be the case that knee height is also highly correlated with birth weight and, as height, is influenced by very early conditions, e.g. before birth, as well. To capture these processes a more complex model is needed, one that includes measures of exposure in multiple critical periods thus allowing room for an assessment of time-varying effects on single metrics of nutritional status as well as effects heterogeneity across metrics.

We conclude that even if we cannot attribute *in toto* the estimated effects to mediating paths *only* involving very early conditions, the evidence gathered suggests that these conditions are important contributors to markers of nutritional status.

#### Robustness of findings: Systematic errors, small sample sizes, the role of chance and selection

Although estimates of effects on knee height are, by conventional standards, "statistically significant" (at levels p < .01 or less), we should provide additional empirical evidence to support our inferences. We do this for three related reasons. The first is that the estimates could be contaminated by systematic measurement of errors. The second is that they are based on a small sample from which the model we estimate demand quite a lot. The third is that the results may be due to chance.

*Systematic errors*. We conducted two tests to assess possibility of systematic underestimation of knee height among those exposed to the flu *and* born in areas impacted by the earthquake. First, the distribution of knee height shows no deviant extreme cases and the smallest values in earthquake areas are within 1.5 of a standard deviation. Second, in a more radical test we *ignored* the lowest values of knee height and re-estimated models. To turn estimated effects from worthy of note (p < [.02-.05]) to mundane (p>.05) we need to exclude observations of knee height *below the first quartile* of the distribution, a rather implausible surgery.

*The role of chance*. Most epidemiological and population health research highlights findings on the basis of classic- Fisher criteria, that is, based on *a priori* chosen significance level (say α < .025 in a two-tailed test). The analysis we carried out was couched on this model as we highlighted results that would pass standard statistical tests with α < .025 in two-tailed test. We are saying nothing new when we point out that this type of criterion can be misleading. This is of concern as extreme values of a statistic can be obtained just by chance and are more likely to occur with small samples and demanding models such as ours. To assess this possibility, we pursue two routes: (a) perform a permutation test [61] and (b) compute bounds for false discovery rates [62].

*(a) Permutation test*: we implement permutation tests on estimates of effects obtained in the most complex model that includes third order interaction effects (female exposure x severity x earthquake). The objective is to add some strength to inferences drawn from conventional hypothesis testing, namely, that the effects on knee height (and height) of the size we observe occur with small probabilities, ideally below .05. This exercise suggests that in a permutation repeated 1000 times the coefficient of the most extreme of exposure (in high flu severity and earthquake) would be lower than what we observe between 4 and 9 percent of the time. This is a bit less stringent and provides weaker support than the conventional test statistic (see S3 Fig).

*(b) False discovery rate*: this is the *conditional* probability that if the null hypothesis is rejected, it is erroneously rejected. This quantity is usually quite different from the conventional α as it is a function of α, power, and the true magnitude of effects. Alternative values of these parameters lead to the graph S4 Fig. Given our p-values ([.01-.02]) and approximate power (.50-.60), we conclude that the probability of uncovering effects only by chance is between .10 and .30, hardly a comforting range but quite common in clinical and population studies [62].

*Selection effects*. It is highly likely that the sample of survivors to age 70 is highly selected and that selection could have been a function of nutritional status and, therefore, a partial result of the events of interest. PREHCO is not unlike other survey of elderly people, all of which confront this problem. We argued before that these biases are in all likelihood attenuating estimates of effects. In S1 File we develop simple expressions to evaluate the potential magnitude of these biases and conclude that, under conditions prevailing in Puerto Rico, we might be underestimating effects by as much as 15 to 20 percent.

## Discussion

Is the magnitude of the 1918 flu effects relevant? While empirical findings confirm that flu exposure, in the broadest sense defined here, is associated with markers of early nutritional status, it is unclear whether their magnitude is substantively meaningful. To shed some light on this issue we proceed indirectly and compare predicted changes in individual stature associated with flu exposure with changes in stature throughout the period of mortality decline in Western Europe. Because knee height appears to be the metric most sensitive to exposure, an ideal test would have been to use the estimated changes in knee height we observe here as a result of exposure with those experienced by other populations. Because there are no historical records of knee height, we cannot perform this test. However, we can exploit the fact that knee height is moderately associated with adjusted height (R-squared~.40 in our sample) and still draw tentative inferences by predicting changes in height from estimated changes in knee height. We find that the reduction in height implied by the estimated reduction in knee height due to flu exposure (between 1.5 and 6.0 cms for an average of .89 of the sample's knee height standard deviation) is associated with a *proportionate* adjusted height reduction of about .0243 (based on a log-log regression of adjusted height on knee height). Note that it took forty years, between 1860 and 1900, for the mean height of the Dutch population to experience proportionate gains that are half as large as the losses induced by the flu and earthquake. A similar inference follows from a comparative assessment of the average reduction in height associated with exposure during critical periods in high flu and earthquake severity areas. As an added piece of evidence consider this: an increase of 1 cm in knee height in our sample translates into a decrease in mortality risks above age 75 of the order of 4%. This, in turn, translates into an increase in life expectancy at that age from 5.75 to 6.14, about 7 percent (calculated using life tables for females in Model West of the Coale-Demeny female life table system using levels 7 and 9).

Is the evidence retrieved here strong enough to support the argument the impact of the flu and earthquake may operate outside the narrow window of fetal exposure? We suggested that past research on the long-run effects of the 1918 influenza may be somewhat limited by a preoccupation with fetal exposure. This is justified since there is strong evidence supporting the idea that embryonic and, more generally, intrauterine disruptions are influential. However, fetal development is also about growth of cartilage, bone and muscle tissue, all of which are implicated in subsequent postnatal physical development. Furthermore, impairments in the fetal period can only be aggravated if post-natal conditions are unfavorable, as may happen as a consequence of maternal or paternal illness. This justifies our claim that the flu pandemic could have also perturbed the post-natal period and through both, fetal and postnatal exposures, affected children’s nutritional status.

Our estimates, particularly those for females born in high severity areas and/or in areas impacted by the earthquake-tsunami, are large, statistically "relevant", and robust to checks. This evidence does not imply that fetal exposure is irrelevant but that it, together with postnatal conditions, may combine in a highly poisonous cocktail that impedes attainment of physical growth landmarks.

The paper has shortcomings. First, an ideal test of our hypotheses requires to contrast effects in multiple windows of exposure (fetal, infancy, and early adolescence). Although we did define alternative critical windows (see S1–S4 Tables) model estimation using more than one critical window became quite difficult due data sparsity. Second, while both nutritional status markers are sensitive to the exogenous shocks, one is more so than the other. Unlike knee height, adjusted height is more likely to be influenced by measurement errors and by events that take place in windows of time more removed from infancy and early childhood. It is difficult to tell whether the differential responses of these markers is as a result of errors or due to real differences in physiological processes that underpin development of different parts of the human body. Third, the sample is small and vulnerable to produce effects where there are weak ones. Unlike other research, we are not dealing with observations in the tens of thousands but with an effective sample size orders of magnitude below that. Buffering against this possibility with permutation tests and computations of false discovery rates can only suggest but not prove that our results are immune to false discovery. Fourth, for the most part our results only offer support for effects on females, not on the entire population. Although we propose conjectures that could explain gender differentials, we have no way of testing them with the data at hand. Finally, and on a different note, Puerto Rico is a tiny dot in a world map, with a population size that has always been, then and now, an infinitesimal fraction of the total world population. Why would anybody bother with all of this? It turns out that the unlikely collusion of two simultaneous natural disasters and the accidental availability of empirical records of survivors, generates a unique opportunity to identify effects of broadly defined early exposures to shocks. In particular, we uncovered some evidence suggesting that past research on the impacts of the 1918 flu pandemic may have missed something important: the influence of the combined disruption of fetal and postnatal life on the ultimate fate of subsequent physical growth. We are not so much trumpeting a new finding as we are identifying a relation that deserves a second look in future research on the 1918 pandemic or, importantly, on the long-lasting effects of the COVID19 pandemic.

## Supporting information

S1 Table Definitions of exposure. (PDF)

S2 Table Definitions of categories of exposure. (PDF)

S3 Table Categories associated with three definitions of exposure. (PDF)

S4 Table Final categories of exposure. (PDF)

S1 Fig Flu severity. (PDF)

S2 Fig Adjusted height. (PDF)

S3 Fig Results of a permutation test. (PDF)

S4 Fig Alternative rates of false discovery. (PDF)

S1 File On biases caused by selection due to differential survival. (PDF)

## References

[1] Helgertz J, Bengtsson T. The long-lasting influenza: the impact of fetal stress during the 1918 influenza pandemic on socioeconomic attainment and health in Sweden, 1968–2012. *Demography*. 2019 8 15;56(4):1389–425. 10.1007/s13524-019-00799-x 31325150 PMC6667423

[2] Enserink M. From Two Mutations, an Important Clue About the Spanish Flu. *Science*. 2007;315(5812):582 10.1126/science.315.5812.582 17272689

[3] Chowell G, Simonsen L, Flores J, Miller MA, Viboud C. Death patterns during the 1918 influenza pandemic in Chile. *Emerg Infect Dis*. 2014;20(11):1803–11. 10.3201/eid2011.130632 25341056 PMC4214284

[4] Oeppen J, Farinas DR, Garcia Ferrero S, Villuendas Hijosa B, Castillo Belmonte AB, Vicente Olmo A. Estimating Reproduction Numbers for the 1889–90 and 1918–20 Influenza Pandemics in the city of Madrid. San Miguel, Acores: Universidades dos Acores; 2010.

[5] Mamelund S-E. Effects of the Spanish influenza pandemic of 1918–19 on later life mortality of Norwegian cohorts born about 1900. Oslo, Norway: Department of Economics, University of Oslo; 2003. Contract No.: Memorandum 2003,29.

[6] Chowell G, Viboud C, Simonsen L, Miller MA, Acuna-Soto R. Mortality patterns associated with the 1918 influenza pandemic in Mexico: evidence for a spring herald wave and lack of preexisting immunity in older populations. *J Infect Dis*. 2010;202(4):567–75. 10.1086/654897 20594109 PMC2945372

[7] Johnson NP, Mueller J. Updating the accounts: global mortality of the 1918–1920 "Spanish" influenza pandemic. *Bulletin of Historical Medicine*. 2002;76(1):105–15. 10.1353/bhm.2002.0022 11875246

[8] Helgertz J, Bengtsson T. The Long-Lasting Influenza: The Impact of Fetal Stress During the 1918 Influenza Pandemic on Socioeconomic Attainment and Health in Sweden, 1968–2012. *Demography*. 2019;56(4):1389–425. 10.1007/s13524-019-00799-x 31325150 PMC6667423

[9] Vollmer S, Wojcik J. The Long-Term Consequences of the Global 1918 Influenza Pandemic: A Systematic Analysis of 117 IPUMS International Census Data Sets. Göttingen, Germany: University of Goettingen 2017 01/01.

[10] Azambuja MIR. Spanish flu and early 20th-century expansion of a coronary heart disease-prone subpopulation. *Tex Heart Inst J*. 2004;31(1):14–21. 15061621 PMC387427

[11] Almond D. Is the 1918 Influenza Pandemic Over? Long-Term Effects of In Utero Influenza Exposure in the Post-1940 U.S. *Population Journal of Political Economy*. 2006;114(4):672–712.

[12] Mazumder B, Almond D, Park K, Crimmins EM, Finch CE. Lingering prenatal effects of the 1918 influenza pandemic on cardiovascular disease. *J Dev Orig* *Health Dis* 2010;1(1):26–34. 10.1017/S2040174409990031 PMC2826837 20198106

[13] Government US. Annual Reports of the War Department. Annual Reports. Washington, D.C.: Government Printing Office; 1919 p. 220–24 cm.

[14] Barker DJP, Eriksson JG, Osmond C. Fetal origins of adult diease: strength of effects and biological basis. *Int J Epidemiology*. 2002;31(6):1235–39. 10.1093/ije/31.6.1235 12540728

[15] Barker DJP. Mothers, Babies, and Health in Later Life. 2nd ed. ed. Edinburgh; New York New York: Churchill Livingstone; 1998.

[16] Gluckman P, Hanson M. The Fetal Matrix: Evolution, Development and Disease: Cambridge; 2005.

[17] Bateson P, Gluckman P. Plasticity, Robustness, Development and Evolution: Cambridge; 2011. 10.1093/ije/dyr240 22422456

[18] Gilbert SF, Barresi MJF. Developmental Biology: Oxford University Press; 2019.

[19] Headey D, Heidkamp R, Osendarp S, Ruel M, Scott N, Black R, et al Impacts of COVID-19 on childhood malnutrition and nutrition-related mortality. *The Lancet*. 2020 7 27. 10.1016/S0140-6736(20)31647-0 PMC7384798 32730743

[20] Coutant R, Carel JC, Letrait M, Bouvattier C, Chatelain P, Coste J, et al Short stature associated with intrauterine growth retardation: final height of untreated and growth hormone-treated children. *Journal of Clinical Endocrinology and Metabolism*. 1998;83(4):1070–4. 10.1210/jcem.83.4.4750 9543119

[21] de Wit CC, Sas TCJ, Wit JM, Cutfield WS. Patterns of Catch-Up Growth. *J Pediatr*. 2013;162(2):415–20. 10.1016/j.jpeds.2012.10.014 23153864

[22] Gluckman PD, Hanson MA. The Develpmental Origins of Health and Disease: Cambridge; 2006.

[23] Rosenfeld CS. The Developmental Origins of Health and Diseases (DOHaD) Concept: Past, Present, and Future London: Elsevier; 2016.

[24] Barker DJ, Osmond C, Law CM. The intrauterine and early postnatal origins of cardiovascular disease and chronic bronchitis. *J Epidemiol Community Health*. 1989;43:237–40. 10.1136/jech.43.3.237 2607302 PMC1052843

[25] Martorell R. The nature of child malnutrition and its long-term implications. *Food and Nutrition Bulletin*. 1999;20(3):288–92.

[26] Butz WP, DaVanzo J, Habicht J-P. Family, Community and Program Influences on the Mortality of Malaysian Infants. 1981.

[27] Palloni A, Millman S. Effects of Inter-Birth Intervals and Breastfeeding on Infant and Early Childhood Mortality. *Population Studies*. 1986;40(2):215–36.

[28] Scrimshaw NS, SanGiovanni JP. Synergism of nutrition, infection, and immunity: an overview. *Am J Clin Nutr*. 1997;66(2):464S–77S. 10.1093/ajcn/66.2.464S 9250134

[29] Johansson SR, Mosk C. Exposure, Resistance and Life Expectancy: Disease and Death During the Economic Development of Japan, 1900–1960. *Population Studies*. 1987;41:207–35. 10.1080/0032472031000142776 11621337

[30] Mosley WH, Chen L. An analytical framework for the study of child survival in developing countries. *Population and Development Review*. 1984;10:25–45. PMC2572391 12756980

[31] Meaney M. Maternal care, gene expression, and the transmission of individual differencesin stress reactivity across generations. *Annu Rev Neurosci*. 2001;24:1161–92. 10.1146/annurev.neuro.24.1.1161 11520931

[32] Bentzinger CF, Wang YX, Rudnicki MA. Building muscle: molecular regulation of myogenesis. *Cold Spring Harbor Perspective in Biology*. 2012;4(2). 10.1101/cshperspect.a008342 PMC3281568 22300977

[33] Wu G, Bazer FW, Cudd TA, Meininger CJ, Spencer TE. Maternal nutrition and fetal development. *J Nutr*. 2004;134(9):2169–72. 10.1093/jn/134.9.2169 15333699

[34] Liu Q, Zhou YH, Yang ZQ. The cytokine storm of severe influenza and development of immunomodulatory therapy. *Cell Mol Immuno*. 2016;13(1):3–10. 10.1038/cmi.2015.74 PMC4711683 26189369

[35] Silasi M, Cardenas I, Kwon J-Y, Racicot K, Aldo P, Mor G. Viral infections during pregnancy. *Am J Reprod Immunol*. 2015;73(3):199–213. 10.1111/aji.12355 25582523 PMC4610031

[36] More VS. Fever in pregnancy and its maternal and fetal outcomes. *Int J Reprod Contracept Obstet Gynecol*. 2017;6(12):5523–7.

[37] Young AP, Wagers AJ. Pax3 induces differentiation of juvenile skeletal muscle stem cells without transcriptional upregulation of canonical myogenic regulatory factors. *Journal of Cell Science*. 2010;123:2632–9. 10.1242/jcs.061606 20605921 PMC2908050

[38] Kramer MS, Kakuma R. The optimal duration of exclusive breastfeeding: A systematic review. Geneva: WHO; 2002 Contract No.: www.who.int/nut/documents/otimal_durartion_of_exc_bfeeding_review_eng.pdf. 10.1002/14651858.CD003517 11869667

[39] Ballard O, Morrow AL. Human milk composition: nutritents and bioactive factors. Pediatrics Clinic North America. 2013;60(1):49–74. 10.1016/j.pcl.2012.10.002 PMC3586783 23178060

[40] Field C. The immunological components of human milk and their effect on immune developmenty in infants. *J Nutr*. 2005;135(1):1–4. 10.1093/jn/135.1.1 15623823

[41] Scrimshaw N. Nutrition and health from womb to tomb. *Food and Nutrition Bulletin*. 1997;18:1–19.

[42] Scrimshaw NS, Taylor C, Gordon J. The interaction between nutrition and Infection: World Health Organization; 1968 5 15, 2008. 4976616

[43] Moore KL, Persuad TVN. The Developing Human: Clinically Oriented Embryology (8th Ed): Philadelphia: Sanuders; 2008.

[44] Black RE, Allen LH, Bhutta ZA, Caulfield LE, de Onis M, Ezzati M, et al Maternal and child undernutrition: global and regional exposures and health consequences. *Lancet*. 2008;371(9608):243–60. 10.1016/S0140-6736(07)61690-0 18207566

[45] Karbownik K, Wray A. Long-Run Consequences of Exposure to Natural Disasters. *J Labor Econ*. 2019;37(3):949–1007.

[46] Eriksson JG, Kajantie E, Osmond C, Thornburg K, Barker DJ. Boys live dangerously in the womb. *Am J Hum Biol*. 2010 5;22(3):330–5. 10.1002/ajhb.20995 19844898 PMC3923652

[47] Coale AJ, Demeny PG, Vaughan B. Regional model life tables and stable populations. New York: Academic Press; 1983.

[48] Morel M.-F. 1991 The care of children: the influence of medical innovation and medical institutions on infant mortality 1750–1914 In Schofield R., Reher D. and Bideau A. (Eds.), *The Decline in Mortality in Europe* (pp. 196–210). Oxford: Clarendon Press.

[49] Goldman N., Pebley A. R., & Beckett M. (2001). Diffusion of ideas about personal hygiene and contamination in poor countries: evidence from Guatemala. *Social science & medicine*, 52(1), 53–69. 11144917 10.1016/s0277-9536(00)00122-2

[50] Hutt C. 1972 “Sex differences in human development” *Human Development* 15:153–170 10.1159/000271239 5042947

[51] Palloni A, Winsborough HH, Scarano F. Puerto Rico Census Project, 1910. Ann Arbor, MI Inter-university Consortium for Political and Social Research [distributor], 2006-1-16 10.3886/ICPSR04343.v1; 2006.

[52] Palloni A, and Pinto-Aguirre G. "Adult mortality in Latin America and the Caribbean" In *International handbook of adult mortality*, pp. 101–132. Springer, Dordrecht, 2011.

[53] Luk J, Gross P, Thompson WW. Observations on mortality during the 1918 influenza pandemic. *Clin Infect Dis*. 2001;33(8):1375–8. 10.1086/322662 11565078

[54] Clark VS. Porto Rico and its problems. Washington, D.C.: The Brookings Institution; 1930.

[55] McEniry, M. AL.Davila and T.Thompson-Colon, 2006. PREHCO Protocol: An Exercise to Examine Interviewer Measurement Error For Anthropometric Measures. Unpublished Report. Center for Demography and Health of Aging, UW-Madison.

[56] Cline MG, Meredith KE, Boyer JT, Burrows B. Decline of height with age in adults in a general population sample: estimating maximum height and distinguishing birth cohort effects from actual loss of stature with aging. *Human Biology*. 1989;61(3):415–25. 2807265

[57] Metter MB., Cayeux MC, Schaller MD, Soguel L., Piazza G., Chiolero R., 2008 “Stature estimation in critically ill patients.” *Clin Nutr ESPEN*: 3: e84–e88.

[58] Chumlea WC and S. Guo, 1992 “Equations for predicting stature in white and black elderly individuals.” *Gerontology*:M 197 e203. 10.1093/geronj/47.6.m197 1430854

[59] Palloni A. and Guend H. “Stature Predictions Equations for Elderly Hispanics in Latin America by Sex and Ethnic Background.” *Journal of Gerontology*: *Medical Sciences* 2005: 60A(6):804–810. 10.1093/gerona/60.6.804 15983187

[60] Jelenkovic et al 2008 “Associations between birth size and later height from infancy through adulthood: An individual based pooled analysis of 28 twin cohorts participating in the CODA Twins project” *Early Human Development*, 120: 53–60 10.1016/j.earlhumdev.2018.04.004 PMC6532975 29656171

[61] Fisher RA. The Design of Experiments. Edinburgh: Oliver & Boyd 1935.

[62] Colquhoun D. An investigation of the false discovery rate and the misinterpretation of p values. *Royal Society Open Science*. 2014;1(3):140216 10.1098/rsos.140216 26064558 PMC4448847

