# Supplementary material for: Impacts of the 1918 flu on survivors' nutritional status: A double quasi-natural experiment
Source: PLoS One. 2020 Oct 20;15(10):e0232805. doi: 10.1371/journal.pone.0232805 (PMC7575088; doi:10.1371/journal.pone.0232805)
Supplement: S1 Table — (PDF) [file pone.0232805.s001.pdf]

S1 Table. DEFINTIONS OF EXPOSURE

The following tables show assignment to classes of exposure implied by the three definitions considered in the paper (see text)

GRAPHIC REPRESENTATION OF PARTIALLY RESTRICTED AND INCLUSIVE DEFINITIONS OF EXPOSURE

EXPOSURE IN UTERO							
BIRTH MONTH AND YEAR		MONTH AND YEAR OF FLU EXPOSURE		GESTATIONAL MONTH AND YEAR OF EXPOSURE			
Month	Year	Month	Year	Month	Year		Category #
GROUP A=PARTIAL EXPOSURE, TAIL END OF GESTATIONAL PERIOD: CLASS 1 thru 7							
Jun	1918	Jun	1918	9			1
Jul	1918	Jun-Jul	1918	8-9			2
Aug	1918	Jun-Aug	1918	7-9			3
Sep	1918	Jun-Sep		6-9			4
Oct	1918	Jun-Oct		5-9			5
Nov	1918	Jun-Nov		4-9			6
Dec	1918	Jun-Dec		3-9			7
GROUP B= FULL EXPOSURE: CLASS 8 thru 14							
Jan	1919	Jun-Jan	1918-19	2-9	1918-19		8
Feb	1919	Jun-Feb	1918-19	1-9	1918-19		9
Mar	1919	Jul-Mar	1918-19	1-9	1918-19		10
Apr	1919	Aug-Apr	1918-19	1-9	1918-19		11
May	1919	Sep-May	1918-19	1-9	1918-19		12
Jun	1919	Oct-Jun	1918-19	1-9	1918-19		13
July	1919	Nov-Jun	1918-19	2-9	1918-19		14
GROUP C=PARTIAL EXPOSURE , INITIAL TAIL OF GESTATIONAL PERIOD : CLASS 15 thru 21							
Aug	1919	Dec-Aug	1918-19	1-7	1918-19		15
Sep	1919	Jan-Sep	1919	1-6	1919		16
Oct	1919	Feb-Oct	1919	1-5	1919		17
Nov	1919	Mar-Nov	1919	1-4	1919		18
Dec	1919	Apr-Dec	1919	1-3	1919		19
Jan	1920	May-Jan	1919-20	1-2	1919-20		20
Feb	1920	Jun-Feb	1919-20	1	1919-20		21
BREASTFEEDING EXPOSURE							
BORN				MONTH AND YEAR AGE OF BREASTFEEDING EXPOSURE TO FLU			
BIRTH MONTH	BIRTH YEAR			MONTH	YEAR		
GROUP I: PARTIALLY AFFECTED AFTER MONTHS 4-6							
Jul-Sep	1917			10-12	1918		22
Oct-Dec	1917			7-12	1918		23
Jan-Mar	1918			4-12	1918		24
GROUP II: FULLY AFFECTED OR PARTIALLY BUT BEFORE MONTH 6							
Apr-Jun	1918			1-12	1918		25
Jul-Sep	1918			1-11	1918-19		26
Oct-Dec	1918			1-9	1918-19		27
Jan-Mar	1919			1-6	1919		28
Apr-Jun	1919			1-3	1919		29
