# Supplementary material for: Impacts of the 1918 flu on survivors' nutritional status: A double quasi-natural experiment
Source: PLoS One. 2020 Oct 20;15(10):e0232805. doi: 10.1371/journal.pone.0232805 (PMC7575088; doi:10.1371/journal.pone.0232805)
Supplement: S2 Table — (PDF) [file pone.0232805.s002.pdf]

S2 Table: DEFINITIONS OF CATEGORIES OF EXPOSURE

CATEGORIES

**Uterine exposure**	**Breastfeeding exposure**
U1=group A= cells numbered 1-7	B1=group I= cells numbered 22-24
U2=group B=cells numbered 8-14	B2=group II=cells numbered 25-29
U3=group C=cells numbered 15-21	

FREQUENCIES AND DATES OF BIRTH ASSOCIATED WITH CATEGORIES

U1=born on or after June 1918&before Dec 1918	N=56
U2=born on or after Jan 1919 and before Aug 1919	N=58
U3=born on or after Aug 1919 and on or before February 1920	N=26
B1=born on or before Mar 1918	N=51
B2=born after Mar 1918 and before Jun 1919	N=127

OVERLAPS AND CROSS-CLASSIFICATION OF CATEGORIES

U1 overlaps with B1: overlap is born before Mar 1918= Light exposure

U1 overlaps with B2: overlap is between Mar and Dec 1918=heavy exposure

U2 does not overlap with B1

U2 overlaps with B2=overlap is between Jan and Jul 1919= worst exposure

U3 does not overlap with B1 or B2
