# Supplementary material for: Impacts of the 1918 flu on survivors' nutritional status: A double quasi-natural experiment
Source: PLoS One. 2020 Oct 20;15(10):e0232805. doi: 10.1371/journal.pone.0232805 (PMC7575088; doi:10.1371/journal.pone.0232805)
Supplement: S3 Table — (PDF) [file pone.0232805.s003.pdf]

S3 Table: CATEGORIES ASSOCIATED WITH THREE DEFINITIONS OF EXPOSURE.

EXPOSURE IN UTERO COMBINED WITH BREASTFEEDING							
BIRTH DATE		GESTATION EXPOSURE		BREASTFEEDING EXPOSURE			
Month	Year	Gestational month of exposure	Year	Breastfeeding month of exposure	Year		
Jul	1917	-		-	1917		
Aug	1917	-		-	1917		
Sep	1917	-		-	1917		
Oct	1917	-		-	1917		
Nov	1917	-		-	1917		
Dec	1917	-		-	1917		
Jan	1918	-	-	6-12	1918		
Feb	1918	-	-	5-12	1918		
Mar	1918	-	-	4-12	1918		
Apr	1918	-	-	3-12	1918		
May	1918	-	-	2-12	1918		
Jun	1918	Jun (9)	1918	1-12	1918-19		
Jul	1918	Jun-Jul (8-9)	1918	1-12	1918-19		
Aug	1918	Jun-Aug (7-9)	1918	1-11	1918-19		
Sep	1918	Jun-Sep (6-9)	1918	1-10	1918-19		
Oct	1918	Jun-Oct (5-9)	1918	1-9	1918-19		
Nov	1918	Jun-Nov (4-9)	1918	1-8	1918-19		
Dec	1918	Jun-Dec (3-9)	1918	1-7	1918-19		
Jan	1919	Jun-Jan (2-9)	1918-19	1-6	1919		
Feb	1919	Jun-Feb(1-9)	1918-19	1-5	1919		
Mar	1919	Jul-Mar (1-8)	1918-19	1-4	1919		
Apr	1919	Aug-Apr (1-9)	1918-19	1-3	1919		
May	1919	Sep-May (1-9)	1918-19	1-2	1919		
Jun	1919	Oct-Jun(1-9)	1918-19	1	1919		
Jul	1919	Nov-Jun(1-8)	1918-19	-	-		
Aug	1919	Dec-Jun (1-7)	1918-19	-	-		
Sep	1919	Jan-Jun (1-6)	1919	-	-		
Oct	1919	Feb-Jun (1-5)	1919	-	-		
Nov	1919	Mar-Jun(1-4)	1919	-	-		
Dec	1919	Apr-Jun (1-3)	1919	-	-		
Jan	1920	May-Jun (1-2)	1919	-	-		
Feb	1920	Jun (1)	1919	-	-		
