# Supplementary material for: Impacts of the 1918 flu on survivors' nutritional status: A double quasi-natural experiment
Source: PLoS One. 2020 Oct 20;15(10):e0232805. doi: 10.1371/journal.pone.0232805 (PMC7575088; doi:10.1371/journal.pone.0232805)
Supplement: S4 Table — (PDF) [file pone.0232805.s004.pdf]

S4 Table: FINAL CATEGORIES OF EXPOSURE (A through D)

A. Main categories defined according to uterine and breastfeeding exposure

U1=green

U2=dark blue

U3=light blue

B1=dark pink

B2=fuchsia

B. Conventional definition based on fetal exposure

Grey column shows mapping reflecting the conventional definition by quarter of birth in 1919

C. Dummy variables used in paper

dummyF_1=1 if birthyear==1918|birthyear==1919&birthmonth<=6 (Yellow) (Used throughout the paper)

dummyE_1=1 if birthyear==1918& birthmonth>=6 OR birthyear==1919&birthmonth<=10 (Red)

D. Alternative definitions used

a. U1, U2 and U3 as well as B1 and B2 either simultaneously or separately

b. Define U12=1 if U1==1 OR U2==1

c. Define U23=1 if U2==1 or U3==1

d. Define B12=1 if B1==1 OR B2==1

d. Define U123=1 if U1 ==1 or U2==1 or U3==1

f. Define UB=1 if U123==1 OR B12==1

CLASSIFICATION MUNICIOS BY EARTHQUAKE SEVERITY

E. Classificatio of municipios by earthquake severity

Group (i) includes the municipios of Aguada, Aguadilla, Anasco, Isabella and Mayaguez. Group (ii) includes the rest of the West Coast municipios (Cabo Rojo, Hormigueros, Rincon, San Sebastian and Quebradilla). The remaining municipios are in group (iii). This grouping is based on historical accounts of the earthquake-tsunami and is consistent with the geographic location of municipios relative to the epicenter of the earthquake and exposure to the tsunami that accompanied it.
