# Supplementary material for: Impacts of the 1918 flu on survivors' nutritional status: A double quasi-natural experiment
Source: PLoS One. 2020 Oct 20;15(10):e0232805. doi: 10.1371/journal.pone.0232805 (PMC7575088; doi:10.1371/journal.pone.0232805)

S1 Fig. FLU SEVERITY

The severity index is computed as the ratio of the of the observed death rate to the expected one that would be observed if each municipio preserved its own age distribution (from 1910 population census) but had been exposed to the US 1918 age specific mortality rates. For Puerto Rico we use municipio specific age distributions from the micro census samples. For the US we employ age specific death rates estimated by Luk(1). The following is the distribution of the ratio (the vertical red line identifies the third quartile of the distribution)

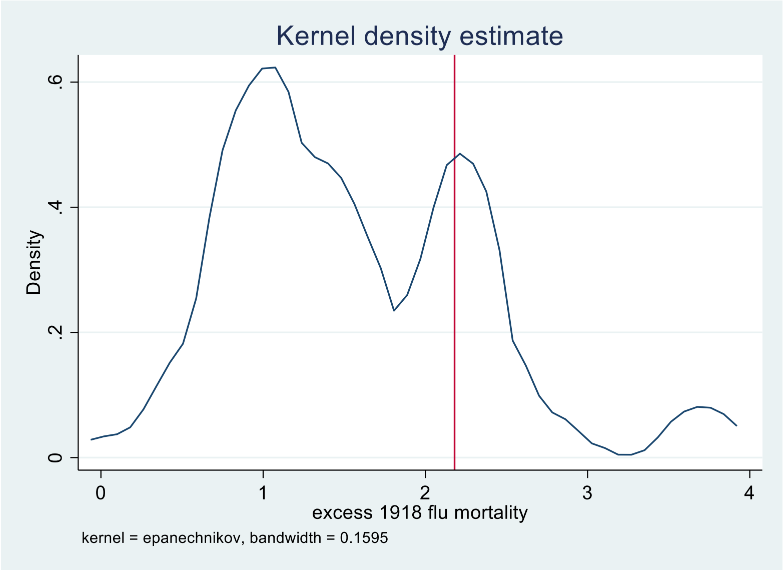

Supplement: S1 Fig — (PDF) [file pone.0232805.s005.pdf]
