# Supplementary material for: Impacts of the 1918 flu on survivors' nutritional status: A double quasi-natural experiment
Source: PLoS One. 2020 Oct 20;15(10):e0232805. doi: 10.1371/journal.pone.0232805 (PMC7575088; doi:10.1371/journal.pone.0232805)
Supplement: S2 Fig — (PDF) [file pone.0232805.s006.pdf]

S2 Fig. ADJUSTED HEIGHT

We use equations relating observed height at older adult ages and a benchmark height measures at young ages. These equations were estimated using the Tucson Epidemiological Study(2) . These longitudinal studies provide means of distinguishing cohort effects from age-related height reductions. Estimates of age-related reductions in this study are very similar to others estimated in Norway, Massachusetts and Wales.

The distribution of differences between adjusted and observed values we obtain in our sample is asymmetric with right skewness. Its median is about 4.35cms and its interquartile range is only 1.75 cms. The graph of the distribution of the differences is below:

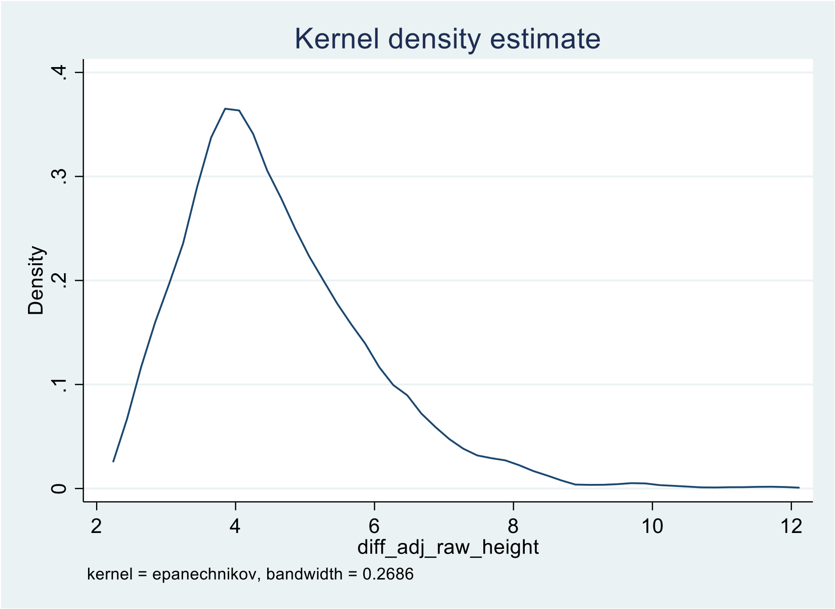

Among females the adjustments are slightly more severe but equally concentrated : the median adjustment is 4.7 cms and the interquartile range is 1.80 cms. These adjustments are quite exaggerated and may overcorrect true values and contract their distribution, thus weakening associations with other variables.
