# Supplementary material for: Impacts of the 1918 flu on survivors' nutritional status: A double quasi-natural experiment
Source: PLoS One. 2020 Oct 20;15(10):e0232805. doi: 10.1371/journal.pone.0232805 (PMC7575088; doi:10.1371/journal.pone.0232805)

**S3 Fig. RESULTS OF A PERMUTATION TEST**

Estimated total effect on knee height= -10.28
Number of replications=1000
Proportion of estimates <= estimated effects(Q)=.07
Standard error of Q=.026
95 % Confidence interval with respect to Q= .04-.10

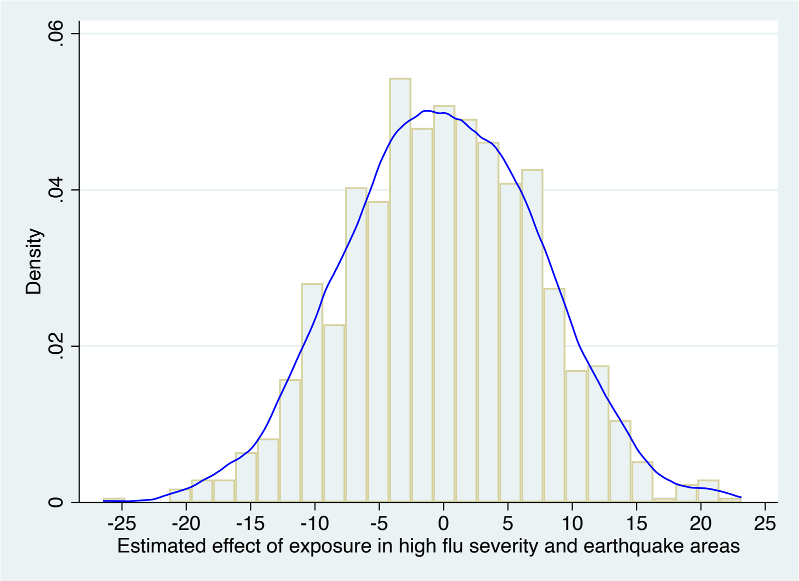

Supplement: S3 Fig — (PDF) [file pone.0232805.s007.pdf]
