# Supplementary figures and images for: Impacts of the 1918 flu on survivors' nutritional status: A double quasi-natural experiment
Source: PLoS One. 2020 Oct 20;15(10):e0232805. doi: 10.1371/journal.pone.0232805 (PMC7575088; doi:10.1371/journal.pone.0232805)

S4 Fig. ALTERNATIVE RATES OF FALSE DISCOVERY

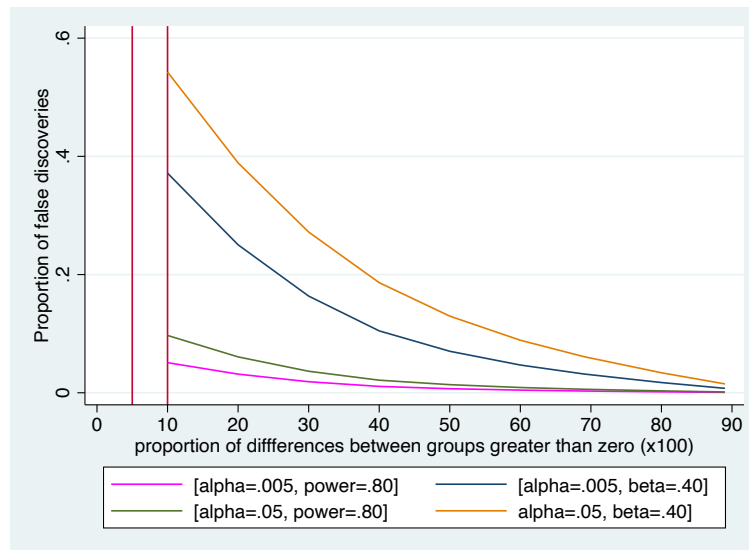

Supplement: S4 Fig — (PDF) [file pone.0232805.s008.pdf]
