# Supplementary material for: Impacts of the 1918 flu on survivors' nutritional status: A double quasi-natural experiment
Source: PLoS One. 2020 Oct 20;15(10):e0232805. doi: 10.1371/journal.pone.0232805 (PMC7575088; doi:10.1371/journal.pone.0232805)
Supplement: S1 File — (PDF) [file pone.0232805.s009.pdf]

S9 On biases caused by selection due to differential survival

As shown below, estimates of the magnitude of selection biases associated with differential survival are a function of multiple parameters most of which are unknown to us. The following derivation is based on two key assumptions. First, the outcome of interest is dichotomous (malnourished or not). Second, the population is homogeneous, that is, individuals can only be classified as exposed or non-exposed and malnourished or non-malnourished.

1.1 The simplest case: population homogeneity

Let B_E be the number of births in the exposed group, π_E the probability of developing malnutrition if exposed and σ_1 the probability of surviving from age 0 to age 70. Similarly, let B_{NE} be the number of births in the non-exposed group, π_{NE} the probability of developing malnutrition and σ_2 the probability of surviving from age 0 to 70 among those non exposed. A measure of the effects of exposure on risks of adult malnutrition is the ratio of the odds of developing malnutrition in the exposed group to the odds of developing malnutrition in the non-exposed group, $OR_T = (\pi_E/(1 \quad \pi_E)/(\pi_{NE}/(1 \quad \pi_{NE}))$. Under the above conditions, the observed odds ratio will be $OR_O = OR_T \ (\sigma_1/\sigma_2)$. It follows that the OR_O is a biased estimate of OR_T and the magnitude of the bias is a function of the ratio σ_1/σ_2. In general, $\sigma_1 < \sigma_2$ and the observed odd ratios will underestimate the true odds ratio.

1.1.1 Mortality homogeneity

To keep things simple, assume that excess mortality among those exposed is experienced mostly during the first year of life and much less there after. Furthermore, assume that morality risks are proportional along the entire age span. Then we can write the probability of surviving from age 0 to age 70 among non exposed as ($exp(-M_0)\ exp(-I_{[1,69]})$) and as ($exp(-\theta M_0) exp(-\delta I_{[1,69]})$) among the exposed. Here M_0 is infant mortality, $I_{[1,69]}$ is the integrated mortality hazard between ages 1 and 70 exactly, and θ and δ are the ratios of infant and adult mortality hazard of the exposed to the non-exposed population. Thus, the ratio of survival probabilities σ_1/σ_2 is given by $exp(-(M_o(\theta-1) + I_{[1,69]}(\delta-1))$ which can be written $exp(- I_{[0,69]} ((M_o/I_{0,69]})(\theta-1) + (I_{[1,69]}/I_{[0,69]})(\delta-1)))$ showing that the final bias depends also on the fraction of all mortality that occurs during infancy. To get a sense for the magnitude of the bias in our case we assume the following: (a) M_o and $I_{[1,60]}$ are approximately equal to the values in the Puerto Rican female life tables of 1920, that is, M_o~.215 and $I_{[1,69]}$ ~1.05 ; (b) θ~1.15, that is, the excess infant mortality is of the order of 15 percent (close to the values estimated to apply to 1918-1919, and (c) δ~1.20, the excess “adult” mortality is about 20 percent. The resulting (downward) bias is

approximately 19 per cent.

1.1.2 Mortality heterogeneity

The above assumes that excess mortality only depends on exposure and not on the occurrence of the event of interest, e.g. malnutrition. To generalize the expression for the magnitude of the bias we assign different mortality levels to those who experience malnutrition and those who do not. We further assume that both infant and "adult" mortality are similarly affected and, finally, that excess mortality is the same irrespective of exposure status and only varies as a function of malnutrition status. The expression for the bias is the following:

$$OR_O/OR_T = (\sigma_{E_1}/\sigma_{E_2})(\sigma_{NE_2}/\sigma_{NE_1})$$

where the first term after the equal sign is the ratio of probabilities of surviving from age 0 to age 70 among exposed individuals who experience malnutrition to those who do not, and the second term after the equal sign is the ratio of survival probabilities among non-exposed individuals who do not experience malnutrition to those who do. Note that the first term is likely to be considerable smaller than 1 whereas the second term will, as a rule, be larger than 1. It follows that, as should be intuitively clear, the downward bias in the odds ratio is likely to be smaller than when mortality homogeneity prevails

In summary, the estimates presented in this paper are surely biased downward but the bias is unlikely to exceed 15 to 20 percent.

1.2 Population heterogeneity

Similar expressions can be derived to represent cases when there is population heterogeneity. For example, we may want to consider the fact that populations are composed of different social classes with heterogeneous risks. Even if exposure is random by social classes, individuals belonging to different social classes could have different propensities to malnutrition both when they are exposed and when they are not exposed, different probabilities of surviving if they become malnourished, and heterogeneus mortality differentials between those who become malnourished and those who do not. These expressions are quite cumbersome and depend on a number of parameters we have little information about. Thus, computing even approximately the magnitude of associated biases is less meaningful than in the cases above where only a few parameters were needed.
